# The European Standard EN 17398:2020 on Patient Involvement in Health Care – a Fairclough-Inspired Critical Discourse Analysis

**DOI:** 10.1177/15271544221088250

**Published:** 2022-03-21

**Authors:** Sigrid Stjernswärd, Stinne Glasdam

**Affiliations:** 1 Department of Health Sciences, 59568 Lund University Faculty of Medicine, Lund, Sweden

**Keywords:** patient involvement, standard, health systems

## Abstract

The concept of ‘patient involvement’ is highlighted in healthcare. However, hindrances can prevent its implementation. This article explored how ‘patient involvement’ is understood and on what ideas this understanding is based through a critical textual analysis of the European document on patient involvement in health systems using a Fairclough-inspired critical discourse analysis. The findings showed that the document arose from a social discourse based on a mix of a neoliberal ideology, with a marketisation of care focusing on a cost-effective and evidence-based logic of care, *and* a humanistic ideology of patient involvement. It had the form of a normative, consensus-based standard, supported by European organisations. The document incorporated a visionary, well-intentioned abstract guide to promote patient involvement across European care contexts, however without addressing hindrances nor differences across the contexts in which it ought to be implemented. It raises questions about its usability, inviting further research into empirical applications.

## Introduction

Throughout the years, Western health policies, educational curricula and institutional health organisations have highlighted patients’ active participation in decision-making in healthcare, which is an element of legal rights movements in many Western countries (Aasen & Dahl, 2018; Elwyn et al., 2020; Glasdam et al., 2015). There is a global movement towards the implementation of a person-centred care model to improve health system performance (Santana et al., 2018). Patient and public involvement (PPI) in research, i.e., research carried out with and by members of the public as opposed to about/to/for them (INVOLVE, 2021), involves patients by building partnerships between the patient and the healthcare professionals in healthcare (Price et al., 2021). Together with the co-production of knowledge, PPI is an ideal in the Western healthcare system, which stipulates that all people should be treated equally and as autonomous individuals with the right to determine their own lives, also in their roles as patients within the healthcare system. Philosophers and healthcare professionals have described patient involvement as an ethical imperative (Elwyn et al., 2013; Madden & Speed, 2017), i.e., an imperative resting on the principle of good clinical practice, respecting the patients’ right to know that their informed preferences should be the basis for professional actions (Elwyn et al., 2013). The consideration of patients’ preferences, in combination with the best available evidence and the clinicians’ experience, is also a pillar within evidence-based medicine (Heiwe et al., 2011; Torgerson & Torgerson, 2008).

Person-centred care (PCC) builds on the idea of placing the patient at the centre of the healthcare system (Pelzang, 2010). The concept of PCC has evolved and been conceptualised through alternative terms (personalised care, relationship-centred care, etc.) and contexts (Santana et al., 2018). In the medical field, the dominating idea is that evidence-based medicine can be translated into practice through person-centred care, which can be facilitated through the use of standards (Masic et al., 2008). A standard is an established measure or model to which other similar things should conform. Standards in healthcare consist of three types: Structure, process, and outcome standards (Miller-Keane, 2003). Structure refers to an evaluation of the setting in which care is rendered and the resources that are available. Process refers to an evaluation of the actual activities carried out by the professionals. Outcome refers to an evaluation of the results of activities in which the professional has been involved in the care/treatment of the patients. Standards of practice take the form of guidelines identifying the content of practice and serve as a model to guide care towards excellence (Miller-Keane, 2003). The underlying philosophy of patient-centred care is that professionals need to understand the patient as a person rather than as a cluster of diseases (Epstein, 2000). Patient-centred care is performed through a range of activities including consideration of the patient's beliefs and values, empathy, and focus on physical, social and emotional needs (McCormack & McCane, 2006; Pelzang, 2010). It means that person-centred care focuses on treating persons receiving healthcare with dignity and respect and involving them in all decisions about their health. However, patient participation is at times presented as activated by health professionals and not by patients themselves. This vision of person–professional interactions thus comes with a risk of erasing the possibility for agency on the part of ‘persons’ and does not evoke collaborative relationships based on mutual respect and equality (Hammell, 2013). Given the potential asymmetries in knowledge and power between patients and professionals, this may be partly predictable. Nevertheless, awareness of such asymmetries by healthcare professionals is essential to facilitate and encourage empowerment and participation originating from patients, based on individual needs and wishes (Glasdam et al., 2020a). The emphasis on considering healthcare users individually is in keeping with person-centred or person-directed care, a (rhetorical) movement away from a paternalistic provision of healthcare in which consumers play an active role in their care (Dahlke & Hunter, 2020; Hammell, 2013).

Even though increased shared-decision making and patient involvement in healthcare are highlighted, challenges pertaining to power inequalities, capabilities to be truly involved at multiple levels of healthcare decisions, and representativeness of those involved, prevail (Glasdam et al., 2015; Ocloo & Matthews, 2016.). If consideration is not given to such challenges and hindrances to active participation and/or collaboration, user/public and patient participation in healthcare and research risks being tokenistic rather than a sign of genuine collaboration (Glover, 2009; Ocloo & Matthews, 2016). Ocloo and Matthews (2016) problematise current models for patient and public involvement in healthcare as they tend to focus on narrow populations and do not properly address power imbalances between the stakeholders, i.e. individual patients and their networks, healthcare professionals on individual and organisational levels, and policy makers. A newly revised European standard on patient involvement has emerged (European Committee for Standardization, 2020), which can serve as an example of a model for patient and public involvement in healthcare, and which represents the empirical material in the current article, exploring ‘patient involvement’. As a prelude to being able to understand an analysis of such a document, the context and some background information regarding the document's origin is necessary. The European Committee for Standardization (CEN), founded in 1961, is a private international non-profit organisation including 34 national members. The CEN collaborates on the development of European standards (ENs) to build and consolidate a European internal market, facilitating the exchange of goods and services throughout this market and positioning Europe on the global market (European Committee for Standardization, 2021a). With the European Committee for Electrotechnical Standardization (CENELEC) and the European Telecommunications Standards Institute (ETSI), the CEN is one of three European standardisation organisations responsible for the development and definition of European standards. As such, the CEN is officially recognised by the European Union (EU) and the European Free Trade Association (EFTA). A European standard “*carries with it the obligation to be implemented at national level by being given the status of a national standard and by withdrawal of any conflicting national standard*”, automatically becoming a national standard in all member countries. Standards are voluntary in the sense that there is no automatic legal obligation to apply them, although laws and regulations may refer to them and make compliance with them compulsory (European Committee for Standardization, 2021b). The CEN provides European standards, which are business driven and developed through consensus-based processes that shall reflect the member countries’ social and economic interests, and lead to increased product safety and quality and to lower transaction costs and prices. The idea is that standards can help promote interoperability, accessibility, sustainability, and the protection of health and safety in workers, consumers and end-users of products and services. The CEN defines a standard as “*a technical document designed to be used as a rule, guideline or definition. It is a consensus-built, repeatable way of doing something*” (European Committee for Standardization, 2021c). There are different types of standards, including requirements and/or recommendations related to products, systems, processes or services, and standards as ways to describe measurements or test methods, or to establish a common terminology within specific sectors (European Committee for Standardization, 2021b). The development of standards involves all interested parties, with over 50,000 technical experts from multiple organisations operating in decentralised, technical groups, coordinated through the CEN's Brussels base. While most standards are initiated by industry, some originate from consumers, small and medium-sized enterprises or associations, or European legislators. Each European standard is automatically recognised and endorsed as the national standard by the 34 European member countries, facilitating the exchange of goods and services on this defined European market. In a global perspective, the CEN describes its role as supportive of European industries’ competitiveness and of international collaboration. The CEN's stakeholders include business and industry, service providers, public authorities and regulators, academia and research centres. Further stakeholders encompass trade associations and a variety of interest groups (European Committee for Standardization, 2021d). Documents of this kind are a product of their time. They mirror a specific historical, social and political context and associated values. Hence, a document about a European standard may reveal something about the context and period during which it was produced, as well as about the healthcare system's organisation. Furthermore, it may reveal something about its creators’ view of the document's recipients, users and beneficiaries, including patients and healthcare professionals, and of humans and the human condition. In order to understand the complexity of such a standard considering the context in which it is produced, the current article's aim is to analyse the European document on patient involvement in healthcare through a critical lens using critical discourse analysis inspired by Fairclough (1993, 2001, 2003, 2005) to explore how ‘patient involvement’ is understood and on what ideas this understanding is based.

## Method

This article is a textual analysis of the European standard on patient involvement in health care (European Committee for Standardization, 2020), that is the English-language version of the Swedish standard version (SIS - Swedish Institute for Standardization, 2020), drawing on Fairclough’s (1993, 2001, 2003, 2005) critical approach to discourse analysis. The overall aim of a Fairclough-inspired analysis is to investigate underlying mechanisms of commonsense understandings of concepts, created through text production and consumption, with the overall purpose of disclosing possible power relationships within a discursive practice. Fairclough (1993) argues that critical discourse analysis can be applied to documentary material as well as texts.

### Empirical Material

The empirical material consists of the Swedish version (SIS - Swedish Institute for Standardization, 2020) of the copyrighted document ‘Patient involvement in health care - Minimum requirements for person-centred care’ (EN 17398:2020) from the European Committee for Standardization (2020). This document was available in both Swedish and English. The English version with a Swedish introduction was used as empirical material in the current study.

### Strategy of Textual Analysis

The concept of discourse covers ways in which the language is used within different domains (Fairclough, 1993). Word choices and ways of compiling texts reveal something about the attitude that exists to what is being written about. Through the use of language people are able to describe experiences and interpretations of reality. Hereby, discourses are closely connected to power as discourses are an important form of social practice that reproduces and transforms knowledge, identities and social relationships. The discourses are shaped by other social practices and structures reproducing or challenging the social order. A critical discourse analysis aims to identify how discursive practice has a role in defining and regulating social practice (Fairclough, 2001, 2003). Fairclough's analysis model includes three levels. The *social practice level* is an analysis of the connection between the discursive practice and the surrounding society where both discursive and non-discursive elements are identified. It also encompasses an analysis of how social practice challenges the existing order of discourse (Fairclough, 1993, 2003). The *discursive practice level* of analysis is an examination of the processes through which the production, distribution, and consumption of texts come into being. These processes are perceived as a form of social practice, forming a link between the text and the social surroundings. The *textual level* of analysis is about capturing how single words and grammatical formulations participate in the construction and reconstruction of social structures (Fairclough, 1993, 2003).

We analysed the text on multiple levels inspired by Fairclough's three-dimensional framework with focus on social practice, discursive practice and text. Initially, we read the text multiple times to grasp how patient-centredness was textually activated. First, the *social practice* analysis focused on the nature of the social practice within which this discourse belonged and which explained why the discourse practice is as it is. Fairclough showed that the theoretical terms ideology and hegemony could be used to theorise changes in power relations in society. Hegemony is understood as power over a society as a whole, where the analysis is about winning consent through ideological means. The discursive changes have their roots in hegemonic struggles. Fairclough understands ideology as the implicit and unconscious materialisation of ideologies that are manifest in individual and collective life, building on the idea that individuals act conditioned by internalised values and ideologies from the surrounding society (Fairclough, 1993, 2003). Second, the focus of the *discourse practice* was on the text's production and distribution. The text's production was in focus through the analysis of ‘interdiscursivity’ and ‘manifest intertextuality’, while its distribution was focused through analysis of ‘intertextual chains’. Further the analysis focused on the text's appearance of ‘coherence’ (Fairclough, 1993). Finally, the focus of the *text analysis* was on the text's linguistic aspects (Fairclough, 1993, 2003). The text was analysed word by word and line by line, with focus on vocabulary, grammar, interactional control and modality. The researchers, with their main schooling rooted in social sciences and nursing (SG) and humanities and nursing (SS), first read and analysed the document separately, then jointly, with a constant dialogue and discussions regarding the analysis process and findings. This dialogue was guided by a Fairclough-inspired lens and questions to the text, lifting the researchers’ potential preconceptions to a theoretical level.

This paper's aim guided the questions posed to the text and to the wider context in which it was created:

- What does the text reveal in terms of the historical, social and political context in which it was created and distributed? (social practice level)
- In which context was the text created and for what purpose(s)? (discursive practice level)
- What does the text reveal in terms of its assumptions about its users and other parties concerned, i.e., society, health organisations, health personnel and patients? (text analysis level)

## Findings

The main findings are presented on the three levels of analysis based on the social practice, the discursive practice and the text analyses, respectively.

### Social Practice

The critical analytical level of social practice focuses on the ideology and the hegemony that could be discerned through the document. The document's ideology was based on a long-standing tradition within healthcare building on patient involvement and person-centred care, which fundamentally challenges the medical logic of the field where diagnoses and treatments are in focus for all activities and encounters in healthcare (Glasdam et al., 2015; Glasdam & Oute, 2019, 2020b). Care services in European countries rest on a neoliberal ideology of care, where modern healthcare is based on a cost-effectiveness rationale. Defining patients as “*partners*” (e.g., p. 5, 11, 19) meant that patients were also responsible for the business and its success. “*Patient involvement*” (pp. 1–27, mentioned 48 times) could otherwise indicate that patients are regarded as customers in meetings with healthcare professionals in the clinic, where they through “*shared decision-making*” (e.g., p. 4, 9, 11) were expected to search for, demand and negotiate the treatment and care they wanted, seek second opinions, try treatments, reject them and try others, have rights, and so on (Glasdam et al., 2015; McLaughlin, 2009; Olesen, 2010). The neoliberal governance discourse was associated with a market discourse where patients are considered as consumers, as Jørgensen and colleagues (2020) also showed. This discourse is grounded in evidence-based logic and the inherently linear structures enable the economy to be managed and to ensure the lowest possible cost level. The neoliberal ideology ensures maximum efficiency and economic profitability. Public employees’ degree of freedom to autonomously make clinical decisions is minimised by using standards and/or division of work into parts, reducing individual influence on own work (Harvey, 2005). It calls for responsibilisation of patients, relatives and healthcare professionals, that is a process whereby citizens are held individually responsible for overseeing and conducting the work that previously would have been the duty of the welfare state (O’Malley, 2009; Teghtsoonian, 2009). Fairclough used the term ‘marketisation of discourse’, based on a social development in late modernity, where market discourse colonises the discursive practices of public institutions (Fairclough, 1993; Phillips & Jørgensen, 2002). This melted biomedical and neoliberal discourses together into one unit (Rose, 2006).

The document was per se a powerful voice in the hegemonic struggles about academisation and professionalisation in healthcare. While acknowledging interprofessionality and specialised knowledge, it simultaneously suggests that all professions work on the basis of a common ground.

Inter-professional team composed of members from the same or different professions and occupations with varied and specialised knowledge, skills and methods, who are committed to a common purpose, approach and performance goals for which they are held mutually accountable (p. 7)

This is a two-way movement in which the various health professions on the one hand are recognised as being unique, and on the other hand, the professions are equalised and equated with the aim of being able to perform the same actions, approaches and services. Nonetheless, the dominant profession, namely medicine, sets the agenda for interprofessional work and collaboration (Glasdam et al., 2020b). This is an extension of the history of nurses, where Foucault (1995) for instance showed that the ‘nurse’ appeared with the birth of the clinic and doctors’ need of assistance to observe patients in the doctors’ absence.

The document both legitimised a humanistic medical and a positivistic medical (research) tradition through the suggested recommendations:

The care personnel shall ensure that the following aspects can be included in the patient narrative:

— the reason why the patient is seeking help or advice, and how their everyday life is affected;

—the patient's feeling of wellbeing;

— the patient's objectives, motivations and values regarding the care process and care outcome. (p. 16)

The professional assessment and guideline-driven care that incorporates evidence-based care and national/local routines (p. 5)

Also, the academisation of other health professions than medicine, e.g., nursing and occupational therapy, has been closely associated with the medical academic tradition of randomised clinical trials as a research method par excellence (Glasdam, 2007). There has been a long-standing struggle in non-medically oriented healthcare professions in terms of being academic professions with/without (partial) detachment from the medical paradigm, and also in terms of belonging to a humanistic scientific position or a medical scientific position (Beedholm & Frederiksen, 2015; Glasdam and Sudmann, 2021; Glasdam et al., 2021; Heyman, 1995).

### Discourse Practice

The document was described as a European standard meant to guide member states to ‘*implement this European Standard*’ (European Committee for Standardization, 2020, p. 3), of which some elements ‘*may be subjects of patent rights*’ (p. 3). The Swedish introduction to the English-language standard was written in ‘we-you’-form with a direct call and appeal to the individual reader to transform the described actions into clinical practice.

“*This standard can help you streamline and quality assure your work. SIS has several services to offer you to facilitate the application of standards in your business* […] *We even offer training, counselling and events around our best-selling standards* […]” (Swedish introduction)

The introduction's ‘we-you’ form puts the sender in a position of being the one who knew and the receiver in a position of being the one who wanted to know. This was comparable with the positions of educator-student and seller-buyer, where the CEN had the positions of both educator and seller of the standard. The document's sender was the European Committee for Standardization (CEN) (‘we’). The document had, expressed as “*shall be given the status of national standard”* (p. 3) in the document, the status of national standard in a number of countries bound to implement this European standard (34 countries), either through its publication or endorsement by these nations. The document's receivers consisted of healthcare and social services (strategic levels) and its employees (employee level), referred to as “*you”* and “*your organisation”* (Swedish introduction), encompassing all from primary care, to home care, preventive care and rehabilitation, but also long-term care and dental practices (p. 4 & Annex A in the document). The document specified ‘*minimum requirements for patient involvement in health care services with the aim to create favourable structural conditions for person-centred care*’ (p. 6). Through the implementation of this standard, the ‘end-consumers’ should be the winners, i.e. the patients and their networks, including family. The overarching aim was to implement standards within the organisation, to streamline work and facilitate quality assurement by creating *“favourable structural conditions for person-centred care*” (p. 6). The aim was to “*facilitate patient involvement and the development of a partnership between the patient and the care personnel*” (p. 4) […] “*before, during and after the actual care that is provided by the care personnel’’* (p. 6*)*. Further, the standardisation could be “*use*[d] *on a strategic level for quality assurance and quality improvement, for procurement, educational and supervisory purposes and as a guiding document for research and development projects in the field of intervention and implementation of person-centred care*” (p. 6). Multiple uses for the document were described, i.e as an aid in planning, managing, implementing and systematically evaluating daily activities; to support patients on a systemic level; as a guide for social care; as an aid in connection with the procurement process of materials and human resources; for education, training and the continuous development of personnel and organisations; and as support pertaining to quality aspects related to patient-involvement (p. 4).

From an *intertextuality* perspective, which placed the current document in a wider context, references were made to Annex A (informative patient cases) and B (information about patient involvement at different healthcare levels and phases), to other standards such as EN 15224 (Swedish Standard Institute, 2017), and to evidence-based care and national/local routines. The document referred to a Cochrane review of central person-centred care (PCC) components, indicating beneficent patient outcomes of PCC in terms of medical outcomes, self-efficacy and self-care. Further, it referred to randomised controlled trials relating to PCC that indicated positive outcomes for both patients and health organisations (e.g., in terms of cost reduction, shorter hospitalisation periods, improved health status and quality of life in end of life care, self-efficacy and disease knowledge, everyday life activities, etc.). Such intertextual references could be interpreted as ‘presuppositions’ in Fairclough's terms (Fairclough, 2003), i.e., the document's receivers were expected to know about these standards and types of trials. The document's bibliography encompassed 31 references, consisting of scientific and health political papers. However, none of them were referred to in the document itself. In the document, it was stated that up-to-date lists and bibliographical references could be obtained on application to the CEN-CENELEC Management Centre or to any CEN member. Lastly, there was an empty page reserved for notes, indicating that the readers could jot down their own reflections in the document. There was a clear *coherence* in terms of visions on health and social care between the sender and the recipient, where the sender reached organisations and professionals with a humanistic perspective on care, quality and professional development, which also included a cost-benefit approach to social and health care.

### Text Analysis

The text structure was visionary and represented a consensus-based, normative European standard for how care professionals ought to practise patient involvement in healthcare through the definition of minimum requirements for person-centred care. Fairclough (1993, 2003) argued that the analysis of words focuses on how individual words reproduced a certain ideology*.* Initially, the document stated that there were no normative references in the text (p. 6). However, the central starting points in the main document were the normative, consensus-based and (self) defined concepts of (*a) patient involvement* and *partnership* between patient and care providers and (b) *patient-centredness/person-centred care (PCC)*. *Patient involvement* and *partnership* between patient and care providers required “*effective interprofessional communication”* (p. 4) and the creation of conditions promoting partnership. Patient involvement was defined as *“patients’ participation in their care on the organisational and/or individual level”* (p. 8). Partnership was defined as “*a relationship of collaboration and mutual respect between a patient and care personnel”* (p. 8). The latter should be based on “*confidentiality”* (p. 10)*, “privacy”* (p. 10)*, “consent”* (p. 4)*, “communication”* (p. 4), *“information sharing”* (p. 12)*, “consensus”* (p. 4)*,* and “*documentation*” (p. 4). The focus of the other normative (self) defined concept of “*patient-centredness/person-centred care (PCC)”* (p. 4) was on patients that were partners and active agents in their own care, self-care and shared decision-making processes. Patients were thus depicted as active and capable subjects, for whose care consideration was taken to their capacities, resources, interests, preferences, goals, knowledge and experience, needs, feelings and wishes. Furthermore, patients’ responsibilities, rights and future plans in a wider societal context ought to be comprehended. PCC is referred to as a “*shared understanding and agreement about what really matters to the patient*” (p. 5) to set care objectives that aligned with the individual patient's understanding of health and quality of life and “*the professional assessment and guideline-driven care that incorporates evidence-based care and national/local routines*” (p. 5). It postulated open communication between patients and care personnel and strategic planning, with consideration taken to patients’ preferences and prerequisites to manage their condition and associated treatments.

Catchwords in the document were 1. *Patients’ narrative and experiences of illness* (p. 9)*,* 2. *Partnership* (p. 11)*, 3. Documentation, care plan and information sharing* (p. 12)*,* and *4. Patient and public management, organisation and decision/policy-making* (p. 13)*.* These catchwords were all normatively described and followed by normative defined requirements for both organisational and clinical levels. Annex A in the document encompassed patient cases, with references made to the contents and requirements described in the main document, and thus the same type of wording. Annex B in the document represented an informative tool, with the description of health care levels and phases at which patient involvement and person-centred care could be operationalised and implemented. It pointed to *“individual*, *operational* and *strategic levels of patient involvement”* (p. 24) and further to different responsibilities and phases in which the partnered patients and care personnel collaborated, including the “*evaluation phase”* (p. 25), the “*allocation phase”* (p. 25), and the *“implementation phase”* (p. 25). Annex B further provided a list of “*resources and tools”* (pp. 26–27) to facilitate patient involvement in person-centred care, which could be reached through weblinks from different institutions active with patient involvement work, such as universities, the World Health Organisation, knowledge centres, and patient organisations.

The document's wording signalled importance, necessity, and hope for successful social and health care. They were expressed in a professional and organisational language in the main document. Annex A in the document, with examples of patient involvement in healthcare, was also formulated in a professional and organisational language. It supported the main aim of patient involvement, of which a main goal was a well-functioning health and social care system defined by low costs, optimal use of resources, and high quality medical care.

The main text was generally written in present and future tense. In line with the document's function of standard and thus of a guiding and binding instrument for the endorsing nations and organisations, several *modal verbs* were used, with a predominance of shall/should and the occasional may, such as exemplified here: (All involved parties) should be taking part, (focus areas) should be taken into account, (patients) may be represented, (care personnel) shall ensure, and (the patient's narrative) could be shared.

“*This* [the patient narrative and an understanding of the meaning of illness for the patient's everyday life] *should be the point of departure for all subsequent interventions in the care of that person*.” (p. 9)

“*In order to facilitate patient involvement in health care services, the following focus areas regarding the patient's narrative and experience of illness, should be taken into account in order to guide the care process:* //” (p. 10).

“*The organisation level shall ensure[….] The care personnel shall ensure that [*…]” (p. 10/p. 11/p. 12/p. 13).

*Reservations* were made in terms of care adjustments demanded by limitations associated with the patient's capabilities and cognitive, emotional and/or functional status. Care should also be provided from the basis of available (and reasonable) care resources. Examples pertaining to patient involvement referred to cases when the patient could not be actively involved at the time e.g., due to limited cognitive capacity. Such situations demanded that care plans should be revised as soon as possible, with involvement of the patient to the best of his/her capacity, whenever possible. Care organisations and personnel should then provide suitable tools to facilitate patient involvement and promote partnerships, such as alternative communication tools, as also illuminated in the patient cases (Annex A in the document).

“*The question “what matters to you” can be a starting point that helps the patients present themselves as persons through a narrative (see Clause A.1). The narrative should in turn build on partnership between the care personnel and patient which encourages and empowers the patient to take part in the care process.* [..] *This* [to understand what an illness means for the patient's everyday life] *can be obtained through a narrative, but also through other means of communication when the patient is not able to provide a narrative.*” (p. 9)

“*In cases where it is not possible to obtain a narrative, an alternative approach is used to capture the information needed*.” (p. 21)

No reservations regarding professionals and organisations were mentioned in the document.

The document acknowledged that culture was at stake in the interactions between professionals and patients.

“*It needs to be recognized that e.g. sensory or cognitive impairments, educational differences, differences in language, or culture can hamper communication between the patient and care personnel*” (p. 10)

However, cultural differences between the 34 countries and their significance for being able to incorporate this standard into their respective health systems were not directly addressed. Implicitly, the document represented an understanding that cultural deviations were divergences that stem from patients, not something related to different countries and their respective health systems.

The document's format mirrored its ‘identity’ of a guiding/binding standard. The sender told the receiver what to do and when. Annex A provided the reader with some tangible examples of how. The sender was thus in control, in its role as a ratified standardisation organisation. So was the document's buyer, e.g., healthcare organisations in member nations, which in turn became senders on organisational levels when/if endorsing the standard. However, there was room for interpretation in terms of how the standard ought to be operationalised and implemented on organisational and especially on individual employee and patient levels, in the specific organisations. This illustrated a kind of interactional control between sender and receiver, where the sender set the tone for what ought to be done to achieve the consensus-based vision of the CEN, and subsequently of what was expected by the member nations and affected organisations.

## Discussion

The current article shows that the European standard document reveals different aspects about the context in which it was created, such as more or less pronounced ideological backdrops, illuminating time and context-specific social and discourse practices, which the document's wordings contribute to uphold. According to Fairclough (2003), the text can be regarded as a part of a social event, and as such, the discussion will focus on a problematisation of the document's currency across different contexts, the actuality of its contents pertaining to patient-involvement and patient-centred care, and of the social practice that it represents.

### The Document's Currency Across Contexts

A key question with standards, which is the subject of recurring debates, relates to their generalisability and applicability across different contexts. The CEN's standards development aims at consolidating a European internal market, facilitating the internal exchange of goods and services, and positioning Europe on the global market (European Committee for Standardization, 2021a). This displays clear economic interests and welfare ambitions on both national and international levels. In that sense, this private international organisation, ratified by the EU and the EFTA, takes on the role of supporting the harmonisation of a standardisation process of health and social care across European countries. This to ultimately promote patient involvement and person-centred care under the direction of the member nations’ health and social care organisations, while also supporting the principles of cost-effectiveness. Simultaneously, the CEN is framed and supported by the neoliberal ideology that rules many of today's Modern states. As neoliberal movements in social and health care are prone to outsource that which can be outsourced (Harvey, 2005), one may ponder whether the CEN is supportive of outsourcing too. Even with a harmonisation of standards, the organisation of the healthcare system and social services and cultural aspects across the member states may differ. It leads to reflection on whether different nations really are comparable across such dimensions, allowing a single standard to be viable in these varied contexts, where the idea is that one standard can be applied to all contexts. In that sense, the CEN implies that the document is generic, i.e., its contents and language allow its application as a minimum standard across all the involved member states without any alterations, although it leaves room for adaptations on levels beyond the minimum requirements. The use of modal verbs and normative definitions of used concepts in the document, for instance, speaks for an instructing, normative approach of that kind. The document's main objectives are for the involved organisations to achieve minimum requirements and structures to facilitate patient involvement and patient-centred care, with an underlying idea that this also equals cost-effective care (Kremer et al., 2020). From the perspective of Fairclough's framework, if efficiency is what healthcare institutions value, healthcare professionals such as nurses who are task-driven to complete medical tasks could be viewed as a model of efficiency (Dahlke & Hunter, 2020). A number of catchwords, or concepts, which can also be recognised in nursing education (Cowling, 2013; Schneider & Ruth-Sahd, 2015), are used to pin down the essence of and means to achieve partnered care through collaboration between personnel and patients, such as the importance of the patient ´s narrative, shared decision-making, information sharing, patient involvement, and person-centred care. In that way, the document adds to the cacophony of blurred and vague conceptual clarifications (Angel & Frederiksen, 2015; Beedholm & Frederiksen, 2019; Madden & Speed, 2017), since the standard represents one concept among others regarding patient involvement and its interpretation is dependent on its recipients. Language reflects power in relationships (Clegg, 1987) and influences how people, for instance healthcare professionals, construct their perceptions of the self and others, and in turn their actions which are based on these perceptions (Beedholm & Frederiksen, 2019; Crowe, 2005). The current study does not allow us to say anything about the recipients’ reception, interpretation and use of the standard. Basically, most people can agree about the fact that individuals are central actors in their own life and disease situations. However, in the vast majority of cases, the interaction between patients and healthcare professionals begins with the fact that a person experiences troubles with body or mind and therefore turns to the health services for help. It is the patient (or relatives) who initially involves healthcare professionals in her/his life whereby a relationship arises, where patients partly transfer the responsibility for their bodies to healthcare professionals. This means that healthcare professionals get the primary responsibility for determining how and how much the patient's bodily and emotional expressions should be emphasised in the specific situations (Glasdam et al., 2020a). The way the current document argues for patient involvement tends to dissolve the patient-transferred responsibility to the professionals and transfer the responsibility for handling the illness and treatment thereof back to the patient, which again raises questions about sense of agency, empowerment, power asymmetries and concepts such as *‘partnership’*. Throughout history, health professionals have been regarded as health experts. Through the concept of ‘patient involvement’, this expert role tends to be shifted towards the patient, away from the professionals.

### The Document's Actuality in Relation to Patient Involvement and Person-Centred Care

The standard's requirements were related to the possibilities to achieve the normatively described ‘person-centred care’ and ‘partnership’ between patients and healthcare professionals. These concepts were based on a notion of the ideal scenario and normative descriptions, not on empirical research on how reality works and is organised. It means that problems with lack of organisational structure, medical priorities, economy, and policies to operationalise ‘patient involvement’ are not taken into consideration in the standard, which is often seen in normative papers, models and theories (Beedholm & Frederiksen, 2019; de Almeida Melo et al., 2020; Paudel et al., 2018). The document has a bibliography with 31 references including scientific papers and health political documents. However, it is not clear how or if these were used or referred to in the European standard as no references appear in the main text. In that light, the document's status comes through as that of an opinion statement, that is a consensus-based normative statement of a vision for social and health care, more than of a standard in line with medical, evidence-based tradition (Heiwe et al., 2011; Torgerson & Torgerson, 2008). In that way, the European Committee for Standardization functions as a professional and political influencer in relation to the European health system generally, politically legitimised by the EU and EFTA. The standard of patient involvement is neither a clinical practice guideline nor a guideline for organisational development. A vision is for the standard to reach all organisational levels within the health system, while at the same time dealing exclusively with patient involvement on an abstract level. It represents all good intentions for the patient as a person at the centre of all health work, while at the same time wanting to streamline and economise the established health system. Further, as presented in the document at an operational level, the idea of patient involvement comes through as being partially similar to asking patients questions about ‘what matters to you’, with focus on the patients’ narratives and what is important for them. This also shows an inclination towards putting the patients in the centre of their care and facilitating involvement and shared decision-making. Examples of this are given in Annex A. Nonetheless, the outcomes of such questions can easily be interpreted and transformed by healthcare professions into actions from the standpoint of ‘what is the matter with you’, which guide their professional, medical actions per se. Such risks are imminent if the necessary structure, resources and organisational climate do not support the translation of person-centred care in theory into practice (Beedholm & Frederiksen, 2019). In line with this, Glasdam and Sudmann (2021) show how the use of various measuring tools in practice acted as a social technology to convert the question ‘what matters to you’ to ‘what is the matter with you’, allowing professionals to become medically action-oriented with focus on solving medical tasks more than focusing on the person behind the diagnosis, the involvement of the patient, and what actually matters to the individual.

### The Document's Actuality in Relation to Social Discourse

The patient is referred to as an active subject and not a passive object; a formulation that goes in line with the ideas of agency and patient empowerment (Abrahamson & Wilson, 2019; Boudioni et al., 2017). This, together with striving for a partnered collaboration between professionals and patients, moves away from paternalistic approaches in healthcare towards participatory approaches (Dahlke & Hunter, 2020). Such a motion is per se positive, as it addresses an inbuilt power imbalance between patients and professionals, at least normatively or in theory. How well theory is operationalised and implemented in practice by healthcare providers to provide patient-centred care is nonetheless debatable, as professionals in practice are subject to power structures within the healthcare institutions, they are in a power position in relation to the patients, and may tend to provide routinised care that does not enable person-centred values (Dahlke & Hunter, 2020; McCormack et al., 2010). With participatory approaches, citizens and patients are expected to take on a more active role in the maintenance and restoration of their health. This requires motivation, health literacy, and in today's digitised society, also digital literacy (Gann et al., 2019; Levin-Zamir & Bertschi, 2018; Svendsen et al., 2020). Not all people are equipped with this, not at all times, and especially not in situations of vulnerability and ill health. At the same time, similar to the concept ‘client-centred’ (Hammell, 2013), the concept ‘person-centred’ is politically expedient from a rhetorical aspect. It may be a professionalising strategy employed to increase professional status and entrench power. The standard settles in the slipstream of the concept ‘patient involvement’ as a floating signifier, pointing to no actual object and with no agreed upon meaning. The concept refers to signs open to interpretation to such an extent that they constitute a floating chain of signifieds, which means that it absorbs rather than emits meaning. The document leaves room for interpretation, contributing to a certain vagueness around the addressed concepts, their operationalisation and implementation on different organisational levels and contexts. The use of floating signifiers is well known in relation to other concepts in healthcare settings, e.g., the concept ‘profession based learning’ (Glasdam, 2008) and ‘reflection’ (Gillet, 2005). In other words, the concept of patient involvement in the current standard document can be understood as empty of tangible meaning, while at the same time implying that all member nations/organisations shall live up to it, i.e., without really knowing what it actually means or what they actually should do as this is up to the individual receivers’ interpretation and operative prerequisites.

### Limitations of the Study

The Fairclough-inspired critical discourse analysis generated knowledge of the discourses on which the standard on patient involvement draws. The critical analysis aimed at illuminating discourses embedded in the document, whether obviously expressed and/or critically addressed in the document's contents or not. The aim was hence not to argue for or against the document, or to develop the standard or clinical practice, the way it was formulated or its implementation as such, but to ‘go behind’ the text and critically analyse embedded discourses. However, this study has some limitations. Texts can be understood in different ways, depending on both the text's actual properties and those of the interpreters (Chouliaraki & Fairclough, 1999). The current article thus offers an interpretation among other possible interpretations. It contributes to a contextualised understanding of a document meant to be endorsed across multiple European nations and thereby illuminates power relationships and drivers at play in this context. However, the analysis can say nothing about the negotiations in the committee that form the basis of this consensus-based standard or the internal power struggles that exist in such a consensus process, which may also be important for understanding the whole complexity of such a standard's construction. Further, the current study cannot answer whether the document is being used and implemented (as expected) by its targeted audience.

## Conclusion

This article explored how ‘patient involvement’ was understood and on what ideas this understanding was based in the European standard on patient involvement in health care (SIS - Swedish Institute for Standardization, 2020). The private, non-profit European Committee for Standardization's document arose from a social discourse based on a mix of a neoliberal ideology, with a marketisation of care focusing on a cost-effective and evidence-based logic of care, *and* a humanistic ideology of patient involvement. This means that the standard is in principle based on two different and opposing ideologies, which may mean that it speaks into the land of all people or the land of no person. It had the form of a normative, consensus-based standard, supported by the EU and EFTA. However, there was no description of how consensus was reached in the group. The document used a lot of catchwords to define patient involvement and added to the cacophony of blurred and vague conceptual clarifications. The document incorporated a visionary, well-intentioned abstract guide to promote patient involvement across European health and social care systems. However, the standard did not openly or directly consider the structural frames of those care systems, and it did not clearly address potential hindrances nor differences across the affected contexts in which the standard is meant to be operationalised and implemented. The current study is a call to management and practitioners in clinical practice to discuss and reflect upon the practical implementation of patient involvement strategies, and the implications of such strategies for patients, relatives and professionals. Further, the results call for awareness about the complexity embedded in the concept of patient involvement in clinical practice, management and healthcare policies. Moreover, the current study gives rise to questions about the standard's usability for the intended users and beneficiaries, inviting further research into empirical applications of the document. Exploring how the current standard is interpreted and implemented in varied contexts and the subsequent implications for patients, relatives, and professions might be of interest.
